# Antiamylase, Antilipase, Antimicrobial, and Cytotoxic Activity of *Nonea obtusifolia* (Willd.) DC. from Palestine

**DOI:** 10.1155/2020/8821319

**Published:** 2020-12-01

**Authors:** Nidal Jaradat, Murad N. Abualhasan, Mohammad Qadi, Linda Issa, Ahmad Mousa, Fathia Allan, Mohammad Hindi, Zaid Alhrezat

**Affiliations:** ^1^Department of Pharmacy, Faculty of Medicine and Health Sciences, An-Najah National University, Nablus 00970, State of Palestine; ^2^Department of Biomedical Sciences, Faculty of Medicine and Health Sciences, An-Najah National University, Nablus 00970, State of Palestine

## Abstract

**Background:**

Medicinal plants are widely used in many cultures, traditions, and civilizations worldwide. Plants with high contents of the valuable biological compounds can efficiently cure many diseases. This study is aimed at assessing, for the first time, the anti-*α*-amylase, antilipase, antimicrobial, and cytotoxic activities of *Nonea obtusifolia* (Willd.) DC. of five extracts from Palestine.

**Methods:**

The antimicrobial activity was estimated using well diffusion method for *N. obtusifolia* plant of five extracts against eight ATCC (American Type Culture Collection) and clinical isolates. The cytotoxic effects for these extracts were evaluated against HeLa (cervical) carcinoma cells using MTS (3-(4,5-dimethylthiazol-2-yl)-5-(3-carboxymethoxyphenyl)-2-(4-sulfophenyl)-2H-tetrazolium) assay. Moreover, the lipase and *α*-amylase inhibitory properties were determined using standard biomedical assays.

**Results:**

The acetone extract of *N. obtusifolia* plant showed a more potent *α*-amylase inhibitory compared with acarbose with IC_50_ values of 25.7 ± 0.08 and 28.18 ± 1.22 *μ*g/ml, respectively. Additionally, the acetone and methanol extracts revealed moderate antilipase activity compared to orlistat with IC_50_ values of 30.19 ± 0.11, 33.11 ± 0.13, and 12.3 ± 0.35 *μ*g/ml, respectively. The methylene chloride extract was found to inhibit the growth of all the tested bacterial and fungal strains and also found to have potential cytotoxic effect against HeLa cancer cell line.

**Conclusion:**

This research work reports for the first time the biological activity of *N. obtusifolia* from Palestine, and the results were promising indicating that *N. obtusifolia* extracts contain valuable bioactive molecules that have a potential anti-*α*-amylase, antilipase, antibacterial, and antifungal cytotoxic potentials. Therefore, *N. obtusifolia* could have a medical significance in the future.

## 1. Introduction

Nowadays, the use of phytomedicine has become an essential type of therapy in our modern life due to its importance in the treatment and prophylaxis of several illnesses [1]. Obviously, their use has existed since ancient civilizations and has been utilized as conventional and complementary types of therapies [2]. Moreover, due to their influence on many systems in the human body, most of the prescribed medicines nowadays are originated from plants [3]. Many studies have shown that most people have consider that natural medicine is safe but they are not aware that their use may be accompanied with many adverse effects, even worse, they may lead to death in some cases [4, 5].

Diabetes mellitus (DM) is a lifelong disease characterized by the inability of the body to metabolize fat, carbohydrate, and lipid which causes hyperglycemia [6]. Recently, diabetes mellitus is considered a worldwide disease, for example, the number of diabetics raised from 180 million in 1980 to 422 million in 2014. In the past two years, it was a leading cause of 1.6 million death, and WHO classified it as the seventh cause of deaths [7–9].

Recently, the search for new natural inhibitors of digestion enzyme from medicinal plants has grown dramatically due to the several side effects of the synthetic inhibitors [10]. Unfortunately, many synthetic drugs have gastrointestinal adverse effects such as diarrhea and abdominal pain. Thus, it is important to investigate new metabolic enzyme inhibitors from a natural origin that have lesser adverse effects [11, 12].

Lipids are essential compounds for all living organisms that function as a building block of the cell membrane and thermal isolators and a source of body energy. In fact, the long-term increase of lipids can cause fat cumulations which leads to obesity causing several types of diseases such as diabetes, hypercholesteremia, and cardiovascular diseases [13]. Actually, any therapeutic agents which can decrease or inhibit the digestion of lipids are of theoretical interest in the treatment of obesity [14].

According to the WHO biostatistical evaluations, obesity became a global health epidemic that is facing humanity today, and approximately 30% of the world population is diagnosed overweight. In addition, obesity is a widespread burden associated with many complications such as cardiovascular, diabetes, and gastrointestinal disorders. Actually, recent investigations on obesity have focused on hyperlipidemia and its role in weight gain. For that, the decreased intake of fat or reduce its absorption by inhibiting the action of lipase enzyme can affect positively in controlling obesity [15, 16].

Microbial infections are considered a worldwide problem and recognized as a threat to the life of humankind. In recent years, antibacterial and antifungal resistance has become an emergent issue in health worldwide. This microbial resistance mainly caused by the misuse of antibiotics. It is necessary to use an alternative method to fight drug resistance problems. People tend to look towards traditional or unconventional methods that are expected to solve the problem and prevent the spreading of infectious diseases [17].

Worldwide, cancer disease continues to be a primary cause of death due to limited and inadequate success of existing treatments for metastatic cancers [18].

In fact, cancer is a group of diseases that develop across time and involve the uncontrolled division of the body cells [19].

One of the fundamental features of cancer is tumor clonality, which means the development of tumors from single cells that begin to proliferate abnormally [20]. This process can be retarded by activities such as apoptosis, cytotoxicity, and antiproliferative activity. An antiproliferative activity is what retards the growth of cells, and so, cancer cells are prevented from multiplying rapidly. Besides, there is cytotoxicity which means causing harm to these rapidly proliferating cells and thereby killing them [21].

*Nonea obtusifolia* (Willd.) DC. (family Boraginaceae) which is an annual herbaceous plant with pointed, hairy and have white bumps leaves. The flowers have a light blue or dark blue color, while the fruit pods are oblong-ovoid, smooth, shiny, and black. However, the flowering period is between January and April months and distributed in the Mediterranean region, specifically in olive tree regions and wet soil. Also, they are common in dry grasslands. The *N. obtusifolia* aerial parts are prepared as porridge are used in the Turkish folk medicine externally to accelerate the healing of wounds and internally to treat the stomach pain [22]. The present research is aimed at determining the effects of *S. multicaulis* five solvent extracts on enzymes like *α*-amylase and lipase as well as aimed at assessing their effect against eight microbial strains and HeLa cancer cells.

## 2. Material and Methods

### 2.1. Plant Collection and Preparation

*N. obtusifolia* leaves were collected in July 2019 from the Mountains of Nablus region of Palestine. The plant was characterized in the Herbal Products Laboratory at An-Najah National University aby Dr. Nidal Jaradat and kept within the voucher specimen code Pharm-PCT-1648.

The leaves of *N. obtusifolia* were washed several times; they were then dried in the shade at ordinary temperature. The drying took about two weeks time until it becomes completely dried, then powdered coarsely, and kept in a well-closed glass jar for further use.

### 2.2. Instrumentation

The instruments used are as follows: rotary evaporator (Heidolph, OB2000, Germany), spectrophotometer-UV-visible (Jenway 7135, England), grinder (Moulinex model, Uno, China), balance (Rad wag, AS 220/c/2, Poland), filter papers (Machrery-Nagel, MN 617 and Whatman no.1, USA), micropipettes (Finnpipette, Finland), incubator (Nuve, Turkey), syringe filter of 0.45 *μ*m pore size (Microlab, China), and microbroth plate (Greiner Bio-One, North America).

### 2.3. Chemicals and Reagents

The chemicals and reagents that used in the current work included petroleum ether and methylene chloride (Alfa-Aesar, England); chloroform and acetone (Sigma Aldrich, Germany); Mueller-Hinton broth (Himedia, India); dimethyl sulfoxide (DMSO) (Riedeldehan, Germany); p-nitrophenyl butyrate, orlistat, and tris-HCl buffer (Sigma-Aldrich, Schnelldorf, Germany); pancreatic lipase type II (Sigma, St. Louis, MO, USA); *α*-amylase (Sigma, Mumbai, India); DNSA (3,5-dinitrosalicylic acid) reagent (Sigma, LA, USA); acarbose (Sigma, St. Louis, USA); and fetal bovine serum, penicillin, streptomycin, and L-glutamine (Sigma, Germany).

### 2.4. Extraction of the Phytochemical Constituents

Twenty grams of the powdered *N. obtusifolia* sample were extracted successively by Soxhlet extractor, according to the method adopted by Abdel-Aal et al. [23], utilizing different organic solvents with analytical reagent quality. These solvents were petroleum ether (40-60°C), methylene chloride (39.6°C), chloroform (61.15°C), acetone (56°C), and finally, methanol (64.7°C). To ensure the complete extraction process, exhaustive extraction was applied with each solvent for 10 h. Extracts of different organic solvents were collected separately into dry clean beakers. Further, they were recovered from the solvents by evaporation in a rotary evaporator at 40°C. The samples were then dried in desiccators for 1 h, the obtained extracts were weighed, and the percentage of each extract was determined.

### 2.5. Antimicrobial

#### 2.5.1. Microbial Isolates

The examined bacterial and fungal isolates were obtained from the American Type Culture Collection (ATCC). The selected species of microorganisms are frequently isolated in clinical settings in our region, and some possess multidrug resistance. The isolates included three Gram-positive isolates: *Enterococcus faecium* (ATCC 700221), methicillin-resistant *Staphylococcus aureus* (MRSA), a clinical strain, and *Staphylococcus aureus* (ATCC 25923), and four Gram-negative strains: *Pseudomonas aeruginosa* (ATCC 27853), *Escherichia coli* (ATCC 25922), *Proteus vulgaris* (ATCC 700221), and *Klebsiella pneumoniae* (ATCC 13883). The used fungal strain was *Candida albicans* (ATCC 90028).

#### 2.5.2. Antimicrobial Test

Five extracts were screened for antimicrobial activity by using the well diffusion method. The bacterial suspension was prepared by picking some colony of overnight agar culture of the test organism and adding it to a test tube containing 5 ml of nutrient broth; then, the turbidity was compared with that of McFarland nephelometer tube no. 0.5 (1.5∗108 cfu/ml); then, it was diluted by taking 1000 *μ*l of suspension, and it was added to 2 ml of nutrient broth (0.5∗108 cfu/ml). The MIC is the lowest concentration of an antimicrobial that inhibits the growth of a microorganism after 18-24 hrs. Each extract was subjected to serial broth dilution technique to determine their minimum inhibitory concentration for all tested microorganisms. Broth microdilution method was used to determine the antimicrobial activity of *N. obtusifolia* five solvents extracts. Each extract of (OA) plant was dissolved in 1 ml DMSO in a concentration of 100% for all solvent extracts (acetone, chloroform, methylene chloride, petroleum ether, and methanol). Then, the final extract concentration was 100 *μ*g/ml. After that, each well was inoculated with microbial inoculums which were prepared in the same medium after dilution of standardized microbial suspension adjusted 0.5 McFarland scale. After well mixing, the 96-well microtitration plates are incubated under 37°C for 24 h. For all bacteria, we tested here, we did four controls including (1) +ve control which contains media+bacteria; (2) -ve control which contains media only; (3) extract control (extract+media): to be sure, there is no contamination and turbidity, and the changes are not occurred due to the plant extract itself (so extracts were serially diluted in this control); (4) DMSO: no DMSO used. The established tests were conducted in triplicates [24].

### 2.6. Porcine Pancreatic Lipase Enzyme Inhibitory Method

E-ach extract of *N. obtusifolia* plant, a working solution (1 mg/ml), was made by dissolving 100 mg of each plant extract in 100 ml of 10% DMSO, then diluted the produced solution to obtain different concentrations (0.05, 0.1, 0.2, 0.3, and 0.4 mg/ml). In addition, a lipase enzyme stock solution (1 mg/ml) was directly prepared before use by dissolving 25 mg of lipase enzyme powder in 25 ml of 10% DMSO then was prepared p-nitrophenyl butyrate (PNPB) stock solution by dissolving 20.9 mg PNPB in 2 ml of acetonitrile. 0.2 ml from each plant extract prepared serial dilutions was mixed with 0.1 ml of lipase enzyme stock solution and completed with Tris-HCl solution to reach 1 ml of volume. Then, incubated for 15 min at 37°C in a water bath, and after 15 min, 100 *μ*l of PNPB solution was added and incubated for 30 min at 37°C. The blank solution was prepared by mixing 100 *μ*l of lipase enzyme (1 mg/ml) solution with a Tris-HCl solution up to 1 ml. Orlistat was used as a positive control and followed the same previous steps as plant fractions. The absorbance was measured utilizing a spectrophotometer (UV-Vis) at 405 nm. However, the lipase enzyme inhibitory potential (*I*%) was measured utilizing the following equation [25]:

(1) $$\begin{array}{c} I\left(\%\right)=\left(\frac{\left[{\text{ABS}}_{\text{blank}}–{\text{ABS}}_{\text{test}}\right]}{\left[\text{ AB}{\text{S}}_{\text{blank}}\right]}\right)*100\%. \end{array}$$

### 2.7. *α*-Amylase Inhibitory Method

Each extract of *N. obtusifolia* plant, a working solution (1 mg/ml), was produced by dissolving 25 mg of each extract in a little amount of 10% DMSO; then, a buffer solution was added up to 25 ml. The solution was then diluted by the buffer to obtain different dilutions (0.01, 0.05, 0.07, 0.1, and 0.5 *μ*g/ml). Later on, an *α*-amylases enzyme working solution (2 units/ml) was prepared by dissolving 12.5 mg of *α*-amylases enzyme powder in a minimum amount of 10% DMSO, and buffer solution was added up to 100 ml. Corn starch solution was prepared by dissolving 1 g of starch in 100 ml distilled water. A 200 *μ*l from each plant extract stock solution was mixed with of 200 *μ*l *α*-amylases stock solution and incubated for 10 min at 30°C in a water bath. After that, 200 *μ*l of corn starch solution was added and incubated for 3 min at 30°C. Moreover, 3, 5-dinitro salicylic acid was added and boiled in a water bath at 85–90°C for 10 min, and after the solution is cooled, was added 5 ml of distilled water. The blank solution was prepared by replacing the plant extract with 200 *μ*l of the buffer. Acarbose was used as a positive control. The absorbance was measured at 540 nm using a UV-Vis spectrophotometer. The *α*-amylase inhibitory potential was calculated using the following formula:

(2) $$\begin{array}{c} I\;\left(\%\right)=\left(\frac{\left[{\text{ABS}}_{\text{blank}}–{\text{ABS}}_{\text{test}}\right]}{\left[\text{ AB}{\text{S}}_{\text{blank}}\right]}\right)*100\%, \end{array}$$

where *I* (%) is the *α*-amylase inhibitory percentage [26].

### 2.8. Cell Culture and Cytotoxicity Assay

HeLa cervical adenocarcinoma cancer cells were cultured in RPMI-1640 media, which was supplemented with 10% fetal bovine serum, 1% penicillin/streptomycin antibiotics and 1% l-glutamine. Cells were grown in a humidified atmosphere with 5% CO_2_ at 37°C. Cells were seeded at 2.6 × 10^4^ cells/well in a 96-well plate. After 48 h, HeLa cells were incubated with various concentrations (6.4, 3.2, 1.6, 0.8, 0.4, 0.2, 0.1, 0.05, 0.025, and 0.0125 mg/ml) of *N. obtusifolia* five extracts for 24 h. Cell viability was assessed by CellTilter 96® Aqueous One Solution Cell Proliferation (MTS) assay according to the manufacturer's instructions (Promega Corporation, Madison, WI). Briefly, at the end of the treatment, 20 *μ*l of MTS solution per 100 *μ*l of media was added to each well and incubated at 37°C for 2 h. The sample absorbance was measured at 490 nm.

### 2.9. Statistical Analysis

Statistical differences were analyzed either with the 2-tailed unpaired Student's *t* test (for comparison between two groups) or one-way analysis of variance (one-way ANOVA with Newman-Keuls' posttests among multiple groups) using Graph Pad Prism 5.0 (GraphPad Software, La Jolla, CA). Data are shown as means ± SD.

## 3. Results and Discussion

Since a very long time, natural products mainly from medicinal plants, minerals, animals, marine products, and microorganisms have been widely used to treat many diseases [27]. There are registers showing medicinal uses of such substances by people from thousands of years before Christ. There are many examples of plant-derived extracts and/or compounds isolated from plants that have been widely used in the treatment of many significant diseases.

There are many advantages of using natural products in drug discovery and development. They represent chemical novelties, and compared with other sources, they can originate lead drug candidates for complex targets. Furthermore, naturally derived constituents possess a chemical diversity unmatched by any synthetic chemical collection, and they can possess bi- and tri-dimensional complex structures, yet be capable of being absorbed and metabolized in the body.

### 3.1. Anti-*α*-Amylase Assessment

Diabetic is a metabolism disorder due to the resistance against insulin or the deficiency of insulin secretion. This metabolic disorder distributed world widely at an alarming rate. Long-term hyperglycemia can cause various complications that affected many cells and organs leading to many lethal diseases [8].

The treatment of diabetes is depending on the oral antidiabetic medications and/or parenteral insulin. Antihyperglycemic medications include biguanides, sulphonylureas, and others such as acarbose [25]. A lot of these medications have serious adverse effects and harmful contraindications. Hereafter, herbal supplements and medications having high therapeutic efficacy with minimal side effects are much favored for patients. The antidiabetic agents from herbals are very promising, and traditionally acclaimed medicinal plants are being investigated for their antidiabetic potential [28]. Nearly 200 species of plant with hypoglycemic properties have been studied [29]. Example of these plants includes the ethanol fruits of cakile maritima scop. (brassicaceae) from Southern Portugal which showed to be 2.19 mmol of equivalent of acarbose [28].

**Figure 1:**
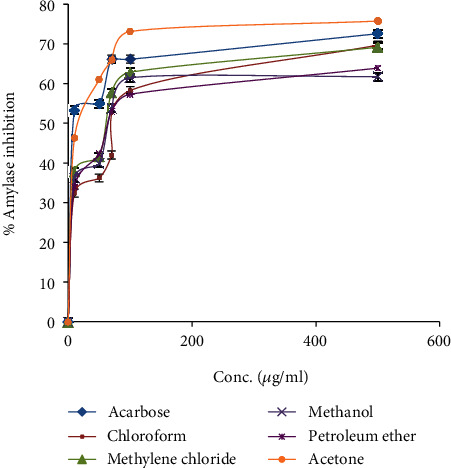
*α*-Amylase inhibitory activity of *N. obtusifolia* five extracts and acarbose drug.

Figure 1 and Table 1 depict the *α*-amylase inhibitory activity and IC_50_ values of *N. obtusifolia* five solvent extracts in comparison with acarbose which used therapeutically in the management of type II of diabetes. Interestingly, all the screened five extracts revealed *α*-amylase inhibitory activity with concentration-dependent manner. However, the acetone fraction of *N. obtusifolia* plant showed potent *α*-amylase inhibitory activity even stronger than the commercial antidiabetic drug acarbose and inhibited *α*-amylase with IC_50_ values of 25.7 ± 0.08 and 28.18 ± 1.22 *μ*g/ml, respectively.

A study conducted by Sarikurkcu et al. found that the methanolic extract of *Anchusa undulata* (the same genus of Nonea) has an *α*-amylase inhibitory activity with amount of 0.193 ± 0.006 mmol ACE/g equivalent of acarbose [30].

Other investigation carried out by Jaradat et al. found that the methanolic extract of *Anchusa ovata* hexane, acetone, methanol, and aqueous extracts have *α*-amylase inhibitory activity with IC_50_ values of 63 ± 1.89, 83.17 ± 0.44, 16.55 ± 1.84, and 72.44 ± 1.86 *μ*g/ml [27].

### 3.2. Antilipase Activity

Antiobesity treatment protocols recommend the regulation of food intake and also intake agents which affect the absorption of dietary fat, metabolism, and storage of fats [31]. Orlistat is a potent inhibitor of gastric and pancreatic lipase, which is a hydrogenated derivative of lipstatin, produced by *Streptomyces toxytricini* and acts by diminishing the absorption of dietary fat. Orlistat forms a covalent bond with the active serine site of lipases and thus inactivates them to hydrolyze dietary fat. It has several side effects including flatulence, incontinence, fecal urgency, fat-soluble vitamin deficiencies, abdominal cramping, steatorrhea, and liquid stools [32].

**Figure 2:**
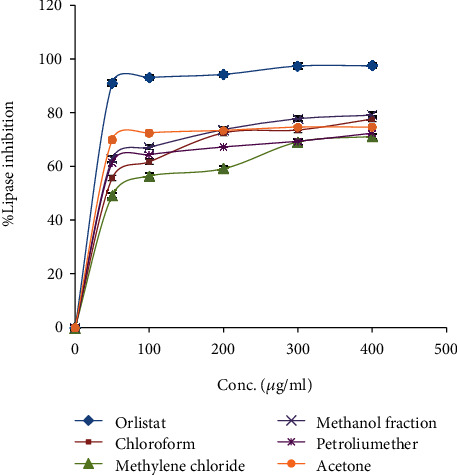
Lipase inhibitory property by orlistat drug and *N. obtusifolia* five extracts.

The porcine pancreatic lipase inhibitory assay was conducted to evaluate the antilipase activity of *N. obtusifolia* five extracts in comparison with the positive control antilipase commercial drug orlistat.  Table 2 and Figure 2 showed that all evaluated extracts have antilipase activity comparing with the positive control orlistat, while the acetone and methanol extracts revealed the highest and potent antilipase effect comparing with orlistat with IC_50_ values of 30.19 ± 0.11, 33.11 ± 0.13, and 12.3 ± 0.35 *μ*g/ml, respectively.

An investigation established by Conforti et al. demonstrated that the *Anchusa azurea* hydroethanolic (70%) extract has lipase inhibitory activity with an IC_50_ value of more than 10 mg/ml [33].

### 3.3. Antimicrobial Effect

In the past two decades, microbial resistance becomes one of the most important prevalent issue all over the world which mainly resulted from overuse or misuse of the antibiotic. In the USA, approximately, 2 million people infected with resistant bacteria yearly. Worldwide, microbial resistance leads annually to 700000 deaths [34].

The broth microdilution assay results showed that not all extracts were effective against the studied microbial strains. In fact, the petroleum ether and acetone extracts did not show ant antimicrobial inhibition effect in the used concentration. However, the chloroform extract inhibited the growth of *S. aureus*, *K. pneumoniae*, *P. vulgaris*, *E. faecium*, and *P. aeruginosa* with MICs of 2.5 mg/ml, respectively. Moreover, methanol extract inhibited the growth of *S. aureus*, *E. faecium*, *P. aeruginosa*, and MRSA with MICs of 12.5, 2.5, 2.5, and 2.5 mg/ml, respectively. Finally, methylene chloride extract inhibited the growth of the screened all microbial strains, particularly against the fungal strain *C. albicans* with MIC of 0.313 mg/ml as presented in Table 3.

The bacteriostatic effect of *Anchusa strigosa* lipid fraction against selected strains of bacterial pathogens has been investigated by Al-Juobory et al. and showed that it was more effective against Gram-positive bacterial strains compared with the Gram-negatives. The antibacterial activity results against Gram-positive strains were in the flowing sequent: *S. faecalis* > *S. aureus* > Bacillus sp., while the effect against Gram-negative was as follows: *P. aeruoginosa* > *Proteus* sp. > *E. coli* > *Enterobacter* sp. > *Klebsiella* sp. [35]. On the other hand, *Anchusa strigosa* essential oil exhibited potent antibacterial activity against both Gram-positive and Gram-negative bacteria, especially in a high concentration (2 and 5 mg/ml) [36].

### 3.4. Cytotoxic Effect

Cytotoxic medications which are commonly known as cytotoxic chemotherapy are the drugs that utilized to kill various types of cancer cells by the inhibition of cell division and in this way cause the death of cancer cells. In addition to the fact that cytotoxic products can be utilized to destroy tumors, they also can boost the outcomes of radiotherapy or surgery and reduce metastases and cancer symptoms [37]. Hela cells were exposed to increasing concentrations of each tested plant sample (6.4, 3.2, 1.6, 0.8, 0.4, 0.2, 0.1, 0.05, 0.025, and 0.0125 mg/ml) for 24 h, and the cell viability was quantified using an MTS assay.

**Figure 3:**
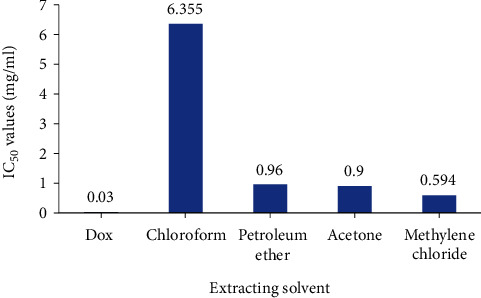
IC_50_ values of *N. obtusifolia* extracts and doxorubicin (Dox) on HeLa cancer cells.

Evaluation of the cytotoxic effect of *N. obtusifolia* five extracts on HeLa cervical adenocarcinoma cell line showed that the methylene chloride extract has the highest IC_50_ value followed by acetone and petroleum ether extracts with IC_50_ values of 0.59, 0.9, and 0.96 mg/ml, respectively, while the methanolic extract of *N. obtusifolia* plant was cytotoxically inactive. However, the methylene chloride extract showed potential cytotoxic activity comparing with the positive control doxorubicin (a potent anticancer drug) which has an IC_50_ value of 0.03 mg/ml (Figure 3).

A study conducted by Sahranavard et al. showed that *Anchusa italica* has a cytotoxic effect against MDBK, WEHI, HepG2, and MCF-7 cancer cell lines with IC_50_ doses more than 100 *μ*g/ml [38].

Moreover, an investigation established by Boskovic et al. reported that *Anchusa officinalis* chloroform, ethyl acetate, ethanol, acetone, and petroleum extracts showed the cytotoxic activity on Hep 2cells with IC_50_ doses of 144.67 ± 2.12, 173.39 ± 0.27, 135.44 ± 0.87, 137.34 ± 1.3, and 176.77 ± 2.26 *μ*g/ml, respectively [39]. Moreover, flavonoid glycosides extracted form viscum albam named as viscumneoside were isolated from the aerial part of *Viscum album*; the cytotoxicity assay showed these compounds have significant inhibitory activities against various cell lines with IC_50_ ≤ 60.00 *μ*mol · L^−1^ [40]. The effects of mistletoe lectin I on the human T-cell leukemia line were investigated with regard to general cell viability and induction of apoptosis. The time- and concentration-dependent direct toxicity towards was determined with IC_50_ values is ranging from 20 to 40 pg/ml [41, 42].

To the best of the author's knowledge, this is the first report of anti-*α*-amylase, antilipase, antimicrobial, and cytotoxic activities of *N. obtusifolia* plant five extracts from Palestine. Further phytochemical and in vivo pharmacological investigations are needed to approve these extraordinary outcomes.

## 4. Conclusion

In summary, this investigation shows that *N. obtusifolia* plant five extracts contain valuable bioactive molecules that acted as anti-*α*-amylase, antilipase, antibacterial, antifungal, and cytotoxic potentials. The acetone extract of *N. obtusifolia* plant illustrated potent *α*-amylase inhibitory comparing with acarbose, and the acetone and methanol extracts revealed potent antilipase activity comparing with orlistat. Moreover, methylene chloride extract inhibited the growth of all the tested bacterial and fungal strains and has potent cytotoxic activity comparing with the positive control doxorubicin. Therefore, *N. obtusifolia* could have a medical significance in the future.

## Figures and Tables

**Table 1:** The *α*-amylase inhibitory activity by acarbose drug and *N. obtusifolia* five extracts in addition to their IC_50_ values (*μ*g/ml).

Concentrations	Acarbose	Chloroform	Methylene chloride	Methanol	Petroleum ether	Acetone
0	0 ± 0	0 ± 0	0 ± 0	0 ± 0	0 ± 0	0 ± 0
10	53.22 ± 1.2	32.37 ± 0.11	37.8 ± 0.02	36.22 ± 0.11	33.97 ± 0.17	46.17 ± 0.17
50	54.91 ± 0.58	36.21 ± 0.11	41.55 ± 0.11	39.85 ± 0.11	42.22 ± 0.25	61 ± 0
70	66.1 ± 1.34	41.98 ± 0.11	57.69 ± 0.22	53.74 ± 0.11	52.99 ± 0.08	65.91 ± 0.08
100	66.1 ± 1.62	58.22 ± 0.11	62.82 ± 0	61.43 ± 0.11	57.29 ± 0.59	73.09 ± 0.08
500	72.54 ± 1.37	69.66 ± 0.11	69.13 ± 0.11	61.75 ± 0	63.87 ± 0.17	75.72 ± 0.08
IC_50_	28.18 ± 1.22	89.12 ± 0.11	53.7 ± 0.09	74.13 ± 0.09	75.85 ± 0.25	25.7 ± 0.08

**Table 2:** The lipase inhibitory activity by orlistat drug and *N. obtusifolia* five extracts and their IC_50_values (*μ*g/ml).

Concentrations	Orlistat	Chloroform	Methylene chloride	Methanol fraction	Petroleum ether	Acetone
0	0 ± 0	0 ± 0	0 ± 0	0 ± 0	0 ± 0	0 ± 0
50	91.05 ± 0.77	55.65 ± 0.14	49.19 ± 0.14	62.8 ± 0.27	61.45 ± 0	69.94 ± 0.09
100	93.1 ± 0.42	61.72 ± 0	56.46 ± 0.14	67.12 ± 0	64.42 ± 0.19	72.5 ± 0.19
200	94.3 ± 0.42	72.5 ± 0.27	59.16 ± 0.41	73.71 ± 0.14	67.25 ± 0.09	73.44 ± 0.09
300	97.4 ± 0.12	73.58 ± 0.27	69.13 ± 0.14	77.76 ± 0.13	69.4 ± 0.07	74.66 ± 0.19
400	97.5 ± 0	77.63 ± 0.27	71.16 ± 0.27	79.11 ± 0.14	72.37 ± 0.09	74.66 ± 0
IC_50_	12.3 ± 0.35	50.11 ± 0.19	63.09 ± 0.22	33.11 ± 0.13	43.65 ± 0.09	30.19 ± 0.11

**Table 3:** Antimicrobial activity MIC values (mg/ml) of *N. obtusifolia* five extracts in a 40 mg/ml concentration.

Plant extracts	*S. aureus*	*E. coli*	*K. pneumoniae*	*P. vulgaris*	*E. faecium*	*P. aeruginosa*	MRSA	*C. albicans*
Chloroform	2.5	R	2.5	2.5	2.5	2.5	R	R
Petroleum ether	R	R	R	R	R	R	R	R
Acetone	R	R	R	R	R	R	R	R
Methylene chloride	1.25	2.5	2.5	2.5	2.5	2.5	2.5	0.313
Methanol	1.25	R	R	R	2.5	2.5	2.5	R

R: resistance.

## Data Availability

No data were used to support this study.
